# Development and Relative Validity of the Chronic Kidney Disease Short Food Frequency Questionnaire (CKD SFFQ) to Determine Diet Quality and Dietary Habits among Adults with Chronic Kidney Disease

**DOI:** 10.3390/nu13103610

**Published:** 2021-10-15

**Authors:** Aljazi Bin Zarah, Mary Carissa Feraudo, Jeanette Mary Andrade

**Affiliations:** 1 Food Science and Human Nutrition Department, University of Florida, Gainesville, FL 32611, USA; aljazibinzarah@ufl.edu (A.B.Z.); mferaudo@ufl.edu (M.C.F.); 2 Community Health Sciences Department, College of Applied Medical Sciences, King Saud University, P.O. Box 10219, Riyadh 11433, Saudi Arabia

**Keywords:** chronic kidney disease, food frequency questionnaire, diet quality

## Abstract

Limited instruments are available to determine diet quality among US adults with chronic kidney disease (CKD). The purpose of this study was two-fold: (1) to develop a food frequency questionnaire, CKD SFFQ, for adults with CKD and (2) to validate the CKD SFFQ against two 24-h recalls in determining diet quality (DQ). A 57-item CKD SFFQ was developed through a content validation method. Adults with CKD (*n* = 46) completed the CKD SFFQ and 2–24-h recalls. Statistical analyses included descriptive statistics, frequencies, *t*-tests, Pearson correlations, and Bland–Altman plots. All data were analyzed using JMP SAS v15 with statistical significance detected at *p* < 0.05. Results showed no differences for the overall DQ (*p* = 0.11) and the nine whole-food components (*p* = 0.07 to *p* = 0.44) when comparing the CKD SFFQ to the 2–24-h recalls. Pearson correlation coefficients ranged from −0.39 (refined grains) to 0.60 (greens and beans). Bland–Altman plots showed overall good agreement and there was a systematic trend towards higher estimates with the CKD SFFQ, particularly for overall DQ, total proteins, and dairy. The majority of participants rarely or never consumed grains, fruits, vegetables, seafood, and plant proteins. The CKD SFFQ was demonstrated to be an acceptable method to determine DQ for adults with CKD.

## 1. Introduction

Chronic kidney disease (CKD) is a slow, progressive disease that affects more than an estimated 37 million or 15% of adults within the United States [1]. CKD has five main stages, which progress at different rates, with the final stage being complete renal failure and the individual is then dependent on dialysis. This progression is dependent upon many factors such as low-grade inflammation, oxidative stress, proteinuria, hypertension, and elevated blood glucose [2,3,4] with the leading contributor being poor diet quality (e.g., high in sodium/saturated fats and processed meats and low consumption of fruits, vegetables, and whole grains) [5,6,7,8].

Diet quality (DQ) is believed to be an influencer in the development and progression of chronic diseases [9]. DQ is a broad concept that refers to how closely a person adheres to dietary guidelines [10]. Essentially, the higher the DQ, the more compliant an adult is in adhering to these guidelines [11]. For those who have CKD, DQ is generally low due to the restrictive nature of the diet that may limit the consumption of fruits, vegetables, and whole grains [11,12,13]. Furthermore, as the disease progresses and reaches the later stages (3–5), protein is restricted to preserve kidney function [12,14,15]. For instance, the protein recommendations for adults in CKD stages 3–5 are 0.28–0.43 g/kg body weight daily with ketoanalogues or 0.55–0.60 g/kg body weight daily without ketoanalogues [14,16]. Considering that a low protein intake may lead to protein-energy malnutrition and cardiac-related death [6,17,18,19], dietary recommendations are for one to consume high-quality protein (e.g., eggs, chicken, red meat). This may lower DQ as, depending on how these foods are prepared, they may be high in saturated fats and sodium [12,20,21].

A method to assess DQ is the Healthy-Eating Index 2015 (HEI-2015). The HEI-2015 is a valid tool that uses a scoring system to determine how closely a dietary pattern adheres to the Dietary Guidelines for Americans 2015–2020 [22]. This has been used to evaluate prospective and cross-sectional correlations between DQ and health outcomes such as risk of CVD mortality [23]. In order to determine DQ based on the HEI-2015 scoring system, dietary intake information needs to be collected [24].

The kidney health professional community uses various methods to monitor an adult’s dietary intake such as 24-h recalls, diet histories, and food frequency questionnaires (FFQ) to provide tailored dietary recommendations [25]. For at least the 24-h recalls and diet histories, these methods may be quite labor intensive and lead to an adult over or underreporting food/beverage intake [26,27,28]. Positively, though, 24-h recalls can provide more accurate estimations of the energy and nutrient intakes to aid in determining the overall DQ for an individual compared to diet histories [29]. In epidemiological and cohort studies, the method generally used to collect dietary intake due to its practicability, affordability and ease of administration is the FFQ [26,27]. A FFQ collects information about the types, quantities and frequency of foods and/or beverages consumed over a predetermined set of time [28]. Even though in large scale studies FFQs are preferred, they are limited by the potential amount of bias and the inability to accurately estimate energy and nutrients consumed to further determine overall DQ. Current studies, though, have been able to determine DQ with FFQ that are similar to DQ derived from 24-h recalls [29,30,31,32]. These studies use various techniques to establish detection of DQ through FFQ such as 7-days of 24-h recalls and 2-FFQs at different time points [30], three 24-h recalls and one FFQ within a three-week period [29], two 24-h recalls and one FFQ [31], or 3–4 days of 24-h recalls and one FFQ [32]. Even though it is recommended that three or four 24-h recalls and one FFQ is necessary for validation of accurate energy and nutrient intake, compliance is often difficult, thus collecting two 24-h recalls and one FFQ in a relatively short time is feasible and may also produce valid measurements [28]. The target audience to validate FFQ to determine DQ has only been on healthy populations with limited focus on chronic disease populations. Therefore, this study is focused on developing a short FFQ that can detect DQ among the CKD population. The purpose of this study was two-fold: (1) to develop a FFQ, CKD SFFQ, for adults with CKD and (2) to validate the CKD SFFQ against two 24-h recalls in determining DQ.

## 2. Materials and Methods

**Figure 1:**
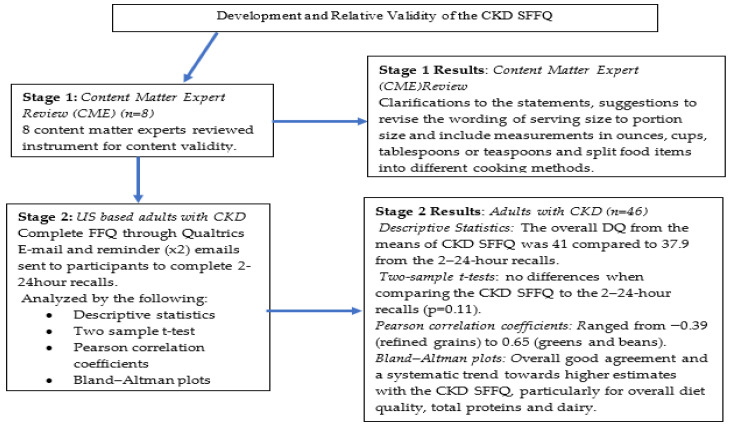
Stages of the study and key findings.

The Chronic Kidney Disease Short Food Frequency Questionnaire (CKD SFFQ) was developed and validated at a Southwestern University. The development and validation followed a two-stage process (Figure 1). Stage one involved the development of the food and beverage items and content matter experts reviewed for content and face validity. In stage two, a criterion validation process was used to determine if the CKD SFFQ measured DQ similar to 2–24-h recalls based on the Healthy Eating Index (HEI)-2015. This study was reviewed and approved by the Institutional Review Board at the University of Florida.

### 2.1. Stage 1: Development of the CKD SFFQ

For the development of this questionnaire, it was based on Affrett and colleagues’ short FFQ [33]. Briefly, the FFQ by Affrett and colleagues was developed to determine dietary intake of the French CKD population and included 49 food and beverage items commonly consumed in France. The instrument had frequency of consuming these items for the past year (never or less than once a month, x times a day, x times a week or x times a month) and portion sizes per food group. For the initial CKD SFFQ, a total of 46 items—38 food items and 8 beverages with serving sizes based on measurements of hand/fingers and cups—were included. Frequency of consuming these foods/beverages was based on the past 30 days to minimize recall bias [16] and based on five categories—daily, 3–5 portions per week, 1–2 portions per week, rarely, or never, which is consistent with other FFQs [34,35,36,37].

#### Design and Participants

For the CKD SFFQ, content matter experts (CMEs) (*n* = 8) reviewed the instrument for content and face validity. The content matter experts were registered dietitian nutritionists who worked in the renal field or general clinical field, had 5–15 years of practice, and had assisted in the development of questionnaires in the past. On a scale from 1 (not at all) to 4 (completely), CMEs rated each statement for clarity, relevance, and ambiguity. Additionally, CMEs provided strengths, weaknesses, and suggestions to enhance each statement. Considering that the rater agreement was high, no additional raters were identified to validate the content of the instrument [38]. CMEs provided suggestions to revise the wording of serving size to portion size and include measurements in ounces, cups, tablespoons, or teaspoons and split food items into different cooking methods, i.e., fried or baked, and added more specific items directed towards an adult who has CKD (e.g., mashed potatoes and roasted baked potatoes). Thus, the total of items included on this questionnaire was 57 items—48 foods and 9 beverages. Supplemental Table S1 summarizes the CKD SFFQ.

### 2.2. Stage 2: Criterion Validation Process

**Figure 2:**
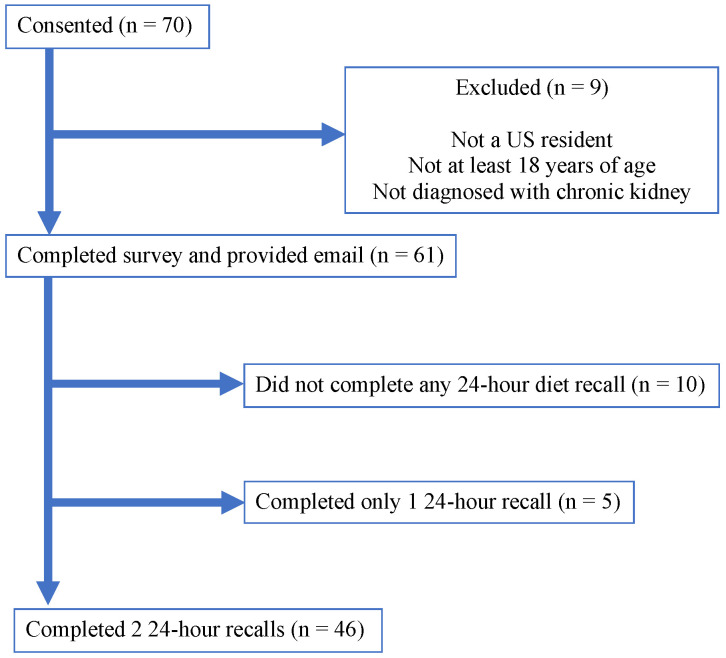
Participation for the CKD SFFQ.

A criterion validation process was used to determine if the CKD SFFQ (57-items) was able to accurately identify participants’ DQ similar to 2–24-h recalls. A priori power analysis [39] was used to estimate the sample size based on the correlation between DQ scores of the CKD SFFQ and 2–24-h recalls using a large effect size of 0.05, an alpha of 0.05, and a power of 95%. A total sample size of 36 participants would be adequate for determining this correlation. Participant recruitment occurred between February 2020–July 2021 with 71 participants initially interested in the study. Sixty-one participants met the inclusion criteria and consented to participate with 46 participants (86.8%) included in the analysis (Figure 2).

Participants were recruited using convenience sampling methods through social media platforms and emails with the headline “Seeking Adults with Kidney Disease for a Pilot Study”. Additionally, participants were recruited at a Southwestern kidney clinic and through ResearchMatch, a national health volunteer registry that was created by several academic institutions and supported by the US National Institutes of Health as part of the Clinical Translational Science Award (CTSA) program. ResearchMatch has a large population of volunteers who have consented to be contacted by researchers for studies that they may be eligible for. Adults were eligible to participate if they were residing within the United States, at least 18 years of age or older, not pregnant or lactating, and had been diagnosed with CKD. Individuals were excluded if they did not meet the above criteria.

Participants who were eligible and consented completed the final version of the CKD SFFQ in Qualtrics (Seattle, WA, USA), an online survey platform. Beyond the CKD SFFQ items, the participants responded to demographic questions (*n* = 7). These demographic questions included sex, age, race/ethnicity, stage of CKD, stage at diagnosis, when diagnosed, and additional health conditions. Before exiting the CKD SFFQ, participants provided their email addresses so that a link to the Automated Self-Administered 24-h Dietary Recall tool (ASA24-2018) with a username and password could be sent to them. The ASA24 is a computerized method to estimate average total energy, macronutrients, micronutrients, and dietary supplement intakes through providing images of foods and beverages for participants to better estimate their portion sizes over a 24-h period [40]. For participants who had limited computer literacy skills, they could complete the 24-h recall via phone call or through a written template. This template included: type of food/beverage consumed, serving size, time consumed, and where they consumed the food. One participant elected to complete the 24-h recall via the telephone with a trained researcher (J.M.A.) who is a registered dietitian nutrition and has experience in collecting these data. Participants (*n* = 25) elected to complete the 24-h recall written template. The researchers (J.M.A. and M.C.F.) reviewed information provided by the participants through the 24-h recall written templates and if further information was needed (e.g., portion sizes or ingredients in the food listed), the researchers would email the participant and ask them these specific questions. Based on the length to complete the 24-h recalls, two participants dropped out from the study and one indicated difficulty with using the ASA24 program and declined to provide information via phone or the written template. Two researchers (A.B.Z. and M.C.F.) entered the information from the phone interview and the paper-based templates into the ASA24-2018 database. Two weeks after the completion of the first 24-h recall, participants were reminded to complete a second 24-h recall. Participants received $10 for completing the CKD SFFQ and $10 for completion of each 24-h recall, for a total potential compensation of $30.

### 2.3. Diet Quality (DQ) Analysis

#### 2.3.1. HEI-2015 Scores

The HEI-2015 was used to determine the individual DQ from the ASA24 for each recorded day. Data from the ASA24 were converted to HEI-2015 scores via JMP SAS v15, using the simple HEI-2015 scoring algorithm method. The HEI-2015 is a valid tool that measures 13 dietary components: total fruits (juices, canned), whole fruits, total vegetables (canned, fresh), greens and beans, whole grains, refined grains, dairy, total protein foods, seafood and plant proteins, sodium, added sugars, fatty acids, and saturated fats compared to the 2015–2020 Dietary Guidelines for Americans [41,42,43]. For at least the food groups, component scores range from 0 to 5 or 0 to 10 and are expressed per 1000 kcal. The specific nutrient scores such as fatty acids are expressed as a ratio of polyunsaturated + monounsaturated fats/saturated fats and added sugars and saturated fats as percentage of energy. For the purposes of this validation process, the scoring from the specific nutrients—added sugars, fatty acids, and sodium—were excluded as the CKD SFFQ focused on whole food consumption as opposed to specific nutrients consumed. Thus, the HEI-2015 ranged from a score of 0–60 as opposed to 0–100. The higher the score, the higher the DQ for a specific component based on the 2015–2020 Dietary Guidelines for Americans [41,44].

#### 2.3.2. CKD SFFQ

The CKD SFFQ was developed to estimate the DQ among whole food components as opposed to specific nutrients—added sugars, fatty acids, and sodium. To align with the HEI-2015, food/beverage items from the CKD SFFQ were categorized into 9 main food components—total fruits (juices, canned, dried), whole fruits, total vegetables (canned, fresh), greens and beans, whole grains, refined grains, dairy, total protein foods, and seafood and plant proteins. The scoring of the CKD SFFQ followed the approach of other FFQ scoring systems [32,45]. Food components that were considered high in sodium (>20% of daily value in a serving size) [46], added sugars (>20% of daily value in a serving size) [47], saturated fat (>20% of daily value in a serving size) [48], and/or low in fiber (<5% of daily value in a serving size) [49], received a score of 0 if they were consumed daily or 3–5 portions/week, a score of 1 if these items were consumed 1–2 portions/week, and dependent on the food item, a score of 1 or 1.5 for consumed rarely or score of 1 or 2 for consumed never. Food components that were low in sodium, added sugars, saturated fat, and/or high in fiber, dependent on the food item, received a score of 2.5, 2, or 1 if they were consumed daily or score of 2, 1.5 or 1 if consumed 3–5 portions/week or a score of 1 if these items were consumed 1–2 portions/week, or a score of 0 for consumed rarely or never. The points were summed up to calculate an overall DQ score that ranged from 0–60 with a higher score reflecting a higher DQ.

### 2.4. Statistical Analysis

Descriptive statistics were used to describe demographics and dietary habits among participants using frequencies for categorical variables and means and standard deviations for continuous variables. Two sample *t*-test assuming equal variances was conducted to detect differences in overall DQ and whole food component scores with the CKD SFFQ and the two 24-h recalls overall and between the two sexes. Pearson’s correlation coefficient analyses and Bland–Altman plots were also performed. The degree of agreement between the CKD-FFQ and the 2–24 h recalls for an individual was assessed by computing the mean ± 2SD (i.e., 95% CI) of the difference. For Pearson’s correlation coefficient, good agreement was ≥0.50, acceptable from 0.20 to 0.49, and poor <0.20 [50,51]. For the Bland–Altman plot outcomes, a good agreement was determined when the difference between the two methods was about one standard deviation of the average DQ scores from the CKD SFFQ and 2–24-h recalls; for fairly good agreement, the difference between the two methods is about two standard deviations; and for poor agreement, the difference between the two methods is three standard deviations [52]. All data were analyzed using JMP SAS v15 with statistical significance detected at a *p* < 0.05.

## 3. Results

### 3.1. Study Participants

Of the 46 participants, the majority were female (69.6%), 70 years of age or older (34.8%), and identified as white/non-Hispanic (80.4%). Participants indicated they were at stage 3 of CKD (70.0%) and the majority were diagnosed more than 5 years ago (65.2%). Furthermore, participants had indicated 4 or more conditions aside from CKD (45.7%) and none were receiving dialysis (Table 1).

### 3.2. Dietary Intake

Two-sample *t*-tests assuming equal variances showed no differences for the overall DQ (*p* = 0.11) and the nine whole-food components (*p* = 0.07 to *p* = 0.44) when comparing the CKD SFFQ to the 2–24-h recalls (Table 2). Comparing sexes, there was a statistical difference in females and males with their overall DQ scores t(21) = −2.31, *p* = 0.02 with females (M = 42.83, SD = 6.73) having higher DQ scores than males (M = 37.07, SD = 8.15).

On average, Pearson’s correlation coefficients (Table 3) in the present study were low, satisfactory correlation coefficients (>0.3) were observed for the estimates of four food groups (44% of the tested food groups): greens and beans, dairy, seafood and plant proteins, and refined grains.

When considering if the methods agreed for individuals, the differences in DQ scores between the CKD SFFQ and the 2–24-h recalls were plotted against the mean DQ scores of the two methods for overall DQ scores and the nine whole food component scores (Supplemental Figure S1). The points are scattered above and below zero in most plots, particularly for total proteins, dairy, and refined and whole grains suggesting that there was no consistent bias of one method compared to the other. For overall DQ, there was some bias towards a positive difference, with a mean difference of 3.2, suggesting that the CKD SFFQ provides higher overall DQ scores compared with the 2–24-h recalls. Similar results were observed for dairy and total proteins. Additionally, there was a trend of decreasing accuracy with increasing overall DQ scores. Furthermore, there was good agreement between seven whole-food components and fair agreement between methods for overall DQ scores, total proteins and refined grains.

**Figure 3:**
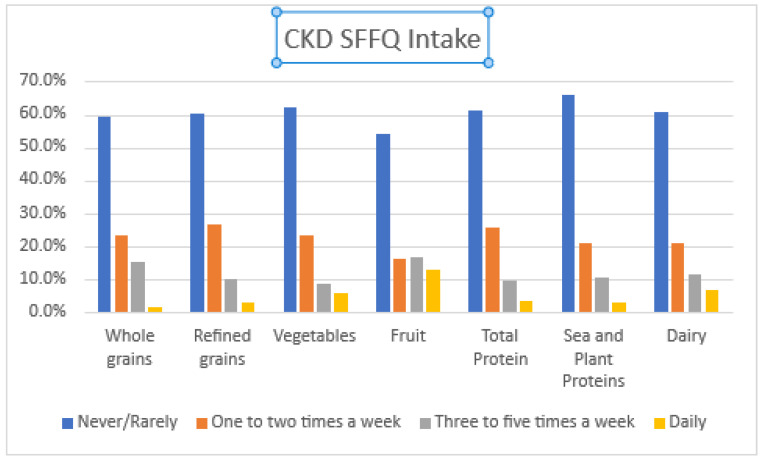
Frequency of consuming whole food components identified through the CKD SFFQ (*n* = 46).

According to the CKD SFFQ, over the past 30 days, the majority of participants rarely or never consumed refined grains (60%), whole grains (59.6%), fruits (54%), vegetables (62.4%), total protein (61.2%), seafood and plant proteins (65.7%), and dairy (60.7%). One to two times per week, participants consumed refined grains (26.7%), total protein (25.6%), vegetables (23.5%), and dairy (21.1%). Few participants consumed refined grains (3.1%), whole grains (1.5%), fruits (13.1%), vegetables (5.6%), total protein (3.7%), seafood and plant proteins (2.8%), and dairy (6.7%) daily. These results are visually displayed in Figure 3.

Even though beverages were not part of the DQ score, participants consumed coffee (43.5%), freshly brewed tea and herb teas (22.2%), and water (91.3%) daily. Participants rarely or never consumed canned/bottled tea or herb teas (86.7%), sweetened beverages (80.4%), artificially sweetened beverages (62.2%), wine (87%), beer (82.6%), or other alcoholic beverages (84.4%).

## 4. Discussion

The study focused on development and relative validity of a short food frequency questionnaire tailored for individuals with chronic kidney disease, CKD SFFQ. The overall results indicated relative validity when comparing the CKD SFFQ to the 2–24-h recalls. The comparison of the CKD SFFQ to the 2–24-h recalls indicates that this tool can adequately provide information for some individual food group consumption amounts (grains, fruits, vegetables, dairy, and protein) and a total DQ score that is similar to other well-developed and validated instruments/approaches. The CKD SFFQ may be an acceptable method to determine DQ on whole-food groups for adults with CKD, which could be used in association with health outcomes and in determining dietary patterns. However, larger scale studies need to be performed.

In this study, the overall mean DQ from the CKD SFFQ was 41.08 compared to the overall mean of 37.89 from the 2–24-h recalls. As the CKD SFFQ is focused on whole food consumption as opposed to determination of added sugars, sodium, and fatty acids, these scores indicated that adults with CKD “needs improvement” in their dietary habits. Further analysis demonstrated that there was a difference between female and male overall DQ scores with females having slightly higher mean scores, 42.8, compared to males, 37.1. This may be due to females consuming more seafood and plant proteins, less total proteins, and less refined grains compared to males. However, caution needs to be applied when interpreting these results due to more females having participated in this study compared to males. Further focus needs to be on validating this tool among sexes and other demographics that may have contributed to these results. Overall, results from the CKD SFFQ DQ showed similarity with another study, in which adults with CKD had low DQ, which indicated that they needed improvements in their dietary habits [11]. Fernandes and colleagues collected 3–24-h recalls from Brazilian adults (*n* = 100) with advanced stages of CKD (3–4) to determine DQ. Median DQ scores were 68.6, which included nutrients such as sodium, fatty acids, and cholesterol. Furthermore, participants had poor diet variety and had the lowest scores (consumed less) in dairy and vegetables, whereas meat/eggs and legumes had the highest scores (consumed more). Considering that the study was conducted in Brazil, where legumes are part of the diet, this may explain why among adults with CKD, legume consumption is high compared to participants in this study.

Limited significant correlations were identified with the CKD SFFQ and the 2–24-h recalls with ranges from −0.52 (refined grains) to 0.60 (greens and beans). Four whole-food components, greens and beans, dairy, seafood and plant proteins, and refined grains, had correlations above 0.3 with the remaining having correlations less than 0.3. The lowest correlation was total proteins at −0.02. This may be related to the large variation between what participants reported on the CKD SFFQ (e.g., rarely/never consumed fried chicken) to the 2–24-h recalls (e.g., consumed fried chicken). As the CKD SFFQ provides a semi-quantitative analysis of dietary intake, it is difficult to measure exact quantities of foods/beverages consumed, thus limiting the ability to provide a more accurate score for the frequency of food/beverage consumed. Instead, this tool provides insight to the types of foods/beverages adults with CKD are consuming on a monthly basis for a more tailored approach to providing nutrition recommendations compared to basing recommendations off 2–24-h recalls.

The Bland–Altman plots showed that the mean difference between the methods for the whole-food component scores, particularly for dairy and total proteins, was positive, suggesting an overestimation of consumption patterns of the CKD SFFQ to the 2–24-h recalls. The higher mean difference in overall DQ scores, 3.2, from the CKD SFFQ was driven mainly by higher estimation of dairy, total proteins, and refined grain scores. This was further confirmed by the weak correlation between the two methods. However, the agreement between the whole food components was good and a fair agreement between the CKD SFFQ and 2–24-h recall overall DQ scores, total proteins, and refined grains.

In this study, 2–24-h recalls were used to determine relative validity among the CKD SFFQ with the HEI-2015 DQ scores. Studies have argued the use of using multiple, 3 or more, 24-h recalls for a more approximate association with dietary intake [53,54,55]. Furthermore, KDOQI guidelines recommend that, for adults with CKD, 3–24-h recalls are sufficient to obtain dietary information [14]. However, recent literature demonstrates that 2–24-h recalls are sufficient to gather dietary intake and reduces burden and dropout among participants [28,56]. Moreover, adults with advanced stages of CKD (3–5) have minimal variability in their diets due to taste aversion [57] or avoiding certain foods/beverages due to nutrient content (e.g., potassium, phosphorus), thus minimal 24-h recalls may be necessary for this population.

From the results of the CKD SFFQ, participants were infrequently consuming whole grains, fruits, vegetables, and plant proteins. Considering that participants (80.41%) were in advanced stages of CKD (3–5), restricting consumption of foods that recommended to prevent further deterioration of kidney status may have been the rationale for avoiding these foods/beverages. Participants (>60%) consumed soft and hard cheeses (1–2 times weekly or more), which, dependent on the serving size and portion, would be lower in phosphorus content compared to milk and yogurt. As information was not collected about if a participant was adhering to a kidney diet or restricting foods based on information obtained from sources, it is difficult to discern the reason participants avoided certain foods/beverages over others. Furthermore, as this tool was established to provide a quick assessment of DQ, the exact nutrient composition of foods/beverages consumed over the past 30 days was not obtained. Thus, it is difficult to know if the rarely/never consumption of these foods/beverages aligned with the KDOQI guidelines. Further modifications of this tool can be done to better estimate the nutrients consumed over the past month.

When focusing on the types of proteins consumed, participants rarely/never consumed plant and/or seafood and instead consumed eggs, baked/grilled chicken and red meat, and homemade protein combination foods (e.g., sandwiches, casseroles) more frequently (3–5 times weekly or daily). Almost half of the participants (40.6%) consumed sausages and other processed meats frequently. Traditionally, protein recommendations for adults with CKD are to consume high quality proteins (e.g., eggs, red meat, chicken) [14,16]. Current studies have indicated that consuming processed and red meats on a frequent basis, at least 2 servings daily, may rapidly progress kidney disease due to the increase in inflammation markers [12,58,59,60], whereas frequent consumption of chicken, eggs, dairy, and fish did not demonstrate progression of this disease [21,59]. Furthermore, recent evidence shows that plant proteins reduce inflammation and slow the progression of this disease [58,61,62,63], even though they contain more phosphorus per serving than meat. Depending on the type of information these participants have been exposed to and if they had other chronic diseases/conditions, it may explain why limited participants consumed plant proteins frequently compared to animal proteins.

The HEI-2015 does not account for beverages aside from those with added sugars. Even though in this study added sugars were not counted in the total DQ, participants consumed water, coffee, and tea daily and rarely or never fruit juice, sugar-sweetened beverages, and alcoholic beverages. A cross-sectional study conducted among Brazilian adults with varying stages of CKD (*n* = 839) also showed that consumption of fruit juice, sugar-sweetened and alcoholic beverages was limited [13]. As participants in this study had a chronic disease such as diabetes or some form of heart disease (32.6% and 67.4%, respectively), it may have accounted for these participants limiting their consumption of sugar-sweetened and alcoholic beverages.

### Strengths and Limitations

The study has several strengths. Even though FFQs exist, none in the US are tailored for those who have CKD. Therefore, the study developed a novel one for health care professionals to use by itself or to rapidly determine DQ in this population. This tool focuses on whole food choices to determine overall DQ. A 24-h recall questionnaire may reflect a range of food intake, but the FFQ examines typical food intake over a longer period.

There are some limitations to this study. Although the sample size was achieved through a power analysis, further validation of this tool with a larger sample size is necessary. As this tool was established to provide a quick assessment of DQ, the exact nutrient composition of foods/beverages was not obtained, thus, this tool can be improved to estimate the nutrients consumed. The majority of participants were white and had advanced stage of CKD, 3. Thus, validating this tool among adults with various races/ethnicities, in the earlier stages of CKD, and on chronic hemodialysis/peritoneal dialysis will be able to show the flexibility with using this tool. As this study began during COVID-19, participants had to complete the CKD SFFQ online and could have the option of completing the 2–24-h recalls online through the ASA24-2018, thus limiting the demographic profile (e.g., those having access to the Internet and who were technology savvy) [64,65]. The researchers had provided a telephone option for participants to complete the 2–24-h recalls, yet less than 4% of the participants opted for it. However, 54.4% of participants preferred completing the 2–24-h recalls through a written template and emailing the completed templates to the researchers. Therefore, providing options to reduce burden on participants may help increase sample size and completion of the study.

## 5. Conclusions

Overall, the use of the developed CKD SFFQ to quickly assess DQ to determine overall foods/beverages consumed among adults with CKD is an acceptable method. The dietary scores obtained through the CKD SFFQ were consistent with those obtained through 2–24-h recalls. The developed CKD SFFQ can be used to promptly assess DQ in adults with CKD. Further research needs to validate this tool among a larger CKD population and in earlier and later stages (e.g., hemo and peritoneal dialysis). The findings from this study may allow clinicians, dietitians, and other healthcare professionals to understand the consumption patterns of particular foods to provide tailored dietary recommendations.

## Figures and Tables

**Table 1:** CKD SFFQ Participants/Demographics (*n* = 46).

Variables	No. of Responses (%)
**Sex**	
Male	14 (30.4%)
Female	32 (69.6%)
**Race/Ethnicity**	
African American	8 (17.4%)
Asian	1 (2.2 %)
Caucasian	37 (80.4%)
**Age**	
18–24 years old	0
25–29 years old	1 (2.2%)
30–49 years old	13 (28.3%)
50–59 years old	8 (17.4%)
60–69 years old	8 (17.4%)
70+ years old	16 (34.7%)
**Length of time with CKD**	
<6 months ago	0
1–2 years ago	7 (15.2%)
3–4 years ago	9 (19.6%)
>5 years ago	30 (65.2%)
**Initial stage of CKD**	
1	6 (13.0%)
2	8 (17.4%)
3	21 (45.7%)
4	6 (13.0%)
5	1 (2.1%)
Don’t know	4 (8.8%)
**Conditions**	
Cancer	5 (10.9%)
Depression	17 (37.0%)
Diabetes	15 (32.6%)
Diverticulosis/Diverticulitis	6 (13.0%)
Gastric reflux	18 (39.1%)
Heart disease (includes high blood pressure, heart attack, artery disease, stroke, angina)	31 (67.4%)
Irritable Bowels	9 (19.6%)
Liver disease	5 (10.9%)
Lung disease	8 (17.4%)
Nausea/Vomiting	9 (19.6%)
Other	28 (60.9%)
Unknown	1 (2.2%)
1 condition	3 (6.5%)
2 conditions	10 (21.7%)
3 conditions	10 (21.7%)
4 or more conditions	21 (45.7%)

**Table 2:** *t*-test: Two sample assuming equal variances (*n* = 46).

Item	HEI-2015		CKD SFFQ		*t*-Test
	Mean	Standard Deviation	Mean	Standard Deviation	
Overall Diet Quality	37.89	62.15	41.08	57.54	0.11
Total Vegetables	3.20	8.57	3.83	1.66	0.09
Greens and Beans	0.76	2.44	0.72	0.74	0.44
Total Fruit	2.36	4.54	2.62	0.50	0.22
Whole Fruit	1.99	4.25	1.62	0.30	0.12
Dairy	3.04	6.20	3.68	2.04	0.07
Total Protein	11.76	56.89	13.48	10.09	0.08
Seafood and Plant Proteins	3.70	12.32	3.05	2.35	0.13
Refined Grains	9.65	25.60	10.71	7.24	0.11
Whole Grains	1.57	2.13	1.37	1.24	0.23

**Table 3:** Pearson rank correlation coefficients between diet quality assessed by CKD SFFQ and diet quality assessed by mean of 2–24 h recalls.

Components	r	(CI 95%) *	*p*-Value **
Overall Diet Quality	0.21	(−0.08–0.46)	0.16
Total Vegetables	0.18	(−0.12–0.44)	0.24
Greens and Beans	0.60	(0.37–0.76)	<0.001 **
Total Fruit	0.23	(−0.12–0.43)	0.17
Whole Fruit	0.21	(−0.15–0.41)	0.16
Dairy	0.41	(0.03–0.55)	0.01 **
Total Protein	−0.02	(−0.30–0.27)	0.91
Seafood and Plant Proteins	0.29	(0.01–0.53)	0.04 **
Refined Grains	−0.52	(−0.52–0.02)	<0.001 **
Whole Grains	0.25	(−0.08–0.46)	0.14

Note. * CI = Confidence interval; ** *p* < 0.05.

## Data Availability

The authors confirm that the data supporting the findings of this study are available within the article and/or its Supplementary Materials.

## Supplementary Materials

The following are available online at https://www.mdpi.com/article/10.3390/nu13103610/s1, Table S1: CKD SFFQ; Figure S1: Bland–Altman plots.

## References

[1] CDC (2019). Chronic Kidney Disease in the United States, 2019. https://www.cdc.gov/kidneydisease/publications-resources/2019-national-facts.html.

[2] Mosnier L.O., Zlokovic B.V., Griffin J.H. (2006). The cytoprotective protein C pathway. Blood.

[3] Mihai S., Codrici E., Popescu I.D., Enciu A.-M., Albulescu L., Necula L.G., Mambet C., Anton G., Tanase C. (2018). Inflammation-Related Mechanisms in Chronic Kidney Disease Prediction, Progression, and Outcome. J. Immunol. Res..

[4] Palmer S.C., Maggo J.K., Campbell K.L., Craig J.C., Johnson D.W., Sutanto B., Ruospo M., Tong A., Strippoli G.F. (2017). Dietary interventions for adults with chronic kidney disease. Cochrane Database Syst. Rev..

[5] USRDS National Institute of Diabetes Digestive Kidney Disease. https://www.usrds.org/media/1730/v2_c05_mortality_18_usrds.pdf.

[6] Nazar C.M.J. (2013). Significance of diet in chronic kidney disease. J. Nephropharmacol..

[7] Gansevoort R.T., Correa-Rotter R., Hemmelgarn B.R., Jafar T.H., Heerspink H.J.L., Mann J.F., Matsushita K., Wen C.P. (2013). Chronic kidney disease and cardiovascular risk: Epidemiology, mechanisms, and prevention. Lancet.

[8] Webster A.C., Nagler E.V., Morton R.L., Masson P. (2017). Chronic Kidney Disease. Lancet.

[9] McCullough M.L., Feskanich D., Stampfer M.J., Giovannucci E.L., Rimm E.B., Hu F.B., Spiegelman D., Hunter D.J., Colditz G., Willett W.C. (2002). Diet quality and major chronic disease risk in men and women: Moving toward improved dietary guidance. Am. J. Clin. Nutr..

[10] Ikizler T.A., Cuppari L. (2021). The 2020 Updated KDOQI Clinical Practice Guidelines for Nutrition in Chronic Kidney Disease. Blood Purif..

[11] Fernandes A.S., Ramos C.I., Nerbass F.B., Cuppari L. (2018). Diet Quality of Chronic Kidney Disease Patients and the Impact of Nutritional Counseling. J. Ren. Nutr..

[12] Kramer H. (2019). Diet and Chronic Kidney Disease. Adv. Nutr..

[13] Santin F., Canella D., Borges C., Lindholm B., Avesani C.M. (2019). Dietary Patterns of Patients with Chronic Kidney Disease: The Influence of Treatment Modality. Nutrients.

[14] Ikizler T.A., Burrowes J.D., Byham-Gray L.D., Campbell K.L., Carrero J.-J., Chan W., Fouque D., Friedman A.N., Ghaddar S., Goldstein-Fuchs D.J. (2020). KDOQI Clinical Practice Guideline for Nutrition in CKD: 2020 Update. Am. J. Kidney Dis..

[15] Goraya N., Wesson D.E. (2015). Dietary interventions to improve outcomes in chronic kidney disease. Curr. Opin. Nephrol. Hypertens..

[16] Levin A., Stevens P.E., Bilous R.W., Coresh J., De Francisco A.L., De Jong P.E., Griffith K.E., Hemmelgarn B.R., Iseki K., Lamb E.J. (2013). KDIGO 2012 Clinical Practice Guideline for the Evaluation and Management of Chronic Kidney Disease. Kidney Disease: Improving Global Outcomes (KDIGO) CKD Work Group. Kidney Int. Suppl..

[17] Kovesdy C.P., Furth S., Zoccali C., World Kidney on Behalf of the World Kidney Day Steering Committee (2017). Obesity and kidney disease: Hidden consequences of the epidemic. Indian J. Nephrol..

[18] Kovesdy C.P., Kopple J.D., Kalantar-Zadeh K. (2013). Management of protein-energy wasting in non-dialysis-dependent chronic kidney disease: Reconciling low protein intake with nutritional therapy. Am. J. Clin. Nutr..

[19] Olokor A.B., Ojogwu I.L., Ugbodaga P.F. (2016). Hyperhomocysteinemia in Chronic Kidney Disease Patients in a Teaching Hospital in Nigeria. Br. J. Med. Med. Res..

[20] Kalantar-Zadeh K., Moore L.W. (2019). Does Kidney Longevity Mean Healthy Vegan Food and Less Meat or Is Any Low-Protein Diet Good Enough?. J. Ren. Nutr..

[21] Lew Q.-L.J., Jafar T.H., Koh H.W.L., Jin A., Chow K.Y., Yuan J.-M., Koh W.-P. (2016). Red Meat Intake and Risk of ESRD. J. Am. Soc. Nephrol..

[22] USDA 2015 Dietary Guidelines|Dietary Guidelines for Americans. https://www.dietaryguidelines.gov/about-dietary-guidelines/previous-editions/2015-dietary-guidelines.

[23] Onvani S., Haghighatdoost F., Surkan P.J., Larijani B., Azadbakht L. (2016). Adherence to the Healthy Eating Index and Alternative Healthy Eating Index dietary patterns and mortality from all causes, cardiovascular disease and cancer: A meta-analysis of observational studies. J. Hum. Nutr. Diet..

[24] Colby S., Zhou W., Allison C., Mathews A.E., Olfert M.D., Morrell J.S., Byrd-Bredbenner C., Greene G., Brown O., Kattelmann K. (2020). Development and Validation of the Short Healthy Eating Index Survey with a College Population to Assess Dietary Quality and Intake. Nutrients.

[25] Beto J.A., Schury K., Bansal V. (2016). Strategies to promote adherence to nutritional advice in patients with chronic kidney disease: A narrative review and commentary. Int. J. Nephrol. Renov. Dis..

[26] Shim J.-S., Oh K., Kim H.C. (2014). Dietary assessment methods in epidemiologic studies. Epidemiol. Health.

[27] Carroll R.J., Midthune D., Subar A.F., Shumakovich M., Freedman L.S., Thompson F., Kipnis V. (2010). Taking Advantage of the Strengths of Two Different Dietary Assessment Instru-ments to Improve Intake Estimates for Nutritional Epidemiology. http://citeseerx.ist.psu.edu/viewdoc/summary?doi=10.1.1.227.6967.

[28] Solbak N.M., Robson P.J., Siou G.L., Al Rajabi A., Paek S., Vena J.E., Kirkpatrick S.I. (2021). Administering a combination of online dietary assessment tools, the Automated Self-Administered 24-Hour Dietary Assessment Tool, and Diet History Questionnaire II, in a cohort of adults in Alberta’s Tomorrow Project. J. Acad. Nutr. Diet..

[29] Procter-Gray E., Olendzki B., Kane K., Churchill L., Hayes R.B., Aguirre A., Kang H.-J., Li W. (2017). Comparison of Dietary Quality Assessment Using Food Frequency Questionnaire and 24-hour-recalls in Older Men and Women. AIMS Public Health.

[30] Newby P.K., Hu F.B., Rimm E.B., Smith-Warner S.A., Feskanich D., Sampson L., Willett W.C. (2003). Reproducibility and validity of the Diet Quality Index Revised as assessed by use of a food-frequency questionnaire. Am. J. Clin. Nutr..

[31] Iqbal R., Ajayan K., Bharathi A.V., Zhang X., Islam S., Soman C.R., Merchant A.T. (2009). Refinement and validation of an FFQ developed to estimate macro- and micronutrient intakes in a south Indian population. Public Health Nutr..

[32] Masip G., Keski-Rahkonen A., Pietiläinen K.H., Kujala U.M., Rottensteiner M., Väisänen K., Kaprio J., Bogl L.H. (2020). Development of a food-based diet quality score and associations with eating styles and nutrient intakes in Finnish twins. Proc. Nutr Soc..

[33] Affret A., Wagner S., El Fatouhi D., Dow C., Correia E., Niravong M., Clavel-Chapelon F., De Chefdebien J., Fouque D., Stengel B. (2017). Validity and reproducibility of a short food frequency questionnaire among patients with chronic kidney disease. BMC Nephrol..

[34] Steinemann N., Grize L., Ziesemer K., Kauf P., Probst-Hensch N., Brombach C. (2017). Relative validation of a food frequency questionnaire to estimate food intake in an adult population. Food Nutr. Res..

[35] Dahl L., Mæland C.A., Bjørkkjær T. (2011). A short food frequency questionnaire to assess intake of seafood and n-3 supplements: Validation with biomarkers. Nutr. J..

[36] Mohammadifard N., Sajjadi F., Maghroun M., Alikhasi H., Nilforoushzadeh F., Sarrafzadegan N. (2015). Validation of a simplified food frequency questionnaire for the assessment of dietary habits in Iranian adults: Isfahan Healthy Heart Program, Iran. ARYA Atheroscler..

[37] Huang Y., Lee M., Pan W., Wahlqvist M.L. (2011). Validation of a Simplified Food Frequency Questionnaire as Used in the Nutrition and Health Survey in Taiwan (NAHSIT) for the Elderly. Asia Pac. J. Clin. Nutr..

[38] Crocker L., Llabre M., Miller M.D. (1988). The Generalizability of Content Validity Ratings. J. Educ. Meas..

[39] Faul F., Erdfelder E., Buchner A., Lang A.-G. (2009). Statistical power analyses using G*Power 3.1: Tests for correlation and regression analyses. Behav. Res. Methods.

[40] Subar A.F., Kirkpatrick S.I., Mittl B., Zimmerman T.P., Thompson E.F., Bingley C., Willis G., Islam N.G., Baranowski T., McNutt S. (2012). The automated self-administered 24-hour dietary recall (ASA24): A resource for researchers, clinicians, and educators from the national cancer institute. J. Acad. Nutr. Diet..

[41] Developing the Healthy Eating Index Developing the Healthy Eating Index (HEI)|EGRP/DCCPS/NCI/NIH. https://epi.grants.cancer.gov/hei/developing.html.

[42] ASA24^®^ Evaluation & Validation ASA24^®^ Evaluation & Validation|EGRP/DCCPS/NCI/NIH. https://epi.grants.cancer.gov/asa24/respondent/validation.html.

[43] Reedy J., Lerman J., Krebs-Smith S.M., Kirkpatrick S.I., Pannucci T., Wilson M.M., Subar A.F., Kahle L.L., Tooze J.A. (2018). Evaluation of the Healthy Eating Index-2015. J. Acad. Nutr. Diet..

[44] Krebs-Smith S.M., Pannucci T.E., Subar A.F., Kirkpatrick S.I., Lerman J.L., Tooze J.A., Wilson M.M., Reedy J. (2018). Update of the Healthy Eating Index: HEI-2015. J. Acad. Nutr. Diet..

[45] Leppälä J., Lagström H., Kaljonen A., Laitinen K. (2010). Construction and evaluation of a self-contained index for assessment of diet quality. Scand. J. Public Health.

[46] FDA: Interactive Food Lable-Sodium. https://www.accessdata.fda.gov/scripts/interactivenutritionfactslabel/sodium.cfm.

[47] FDA: Added Sugars on the New Food Label. https://www.fda.gov/food/new-nutrition-facts-label/added-sugars-new-nutrition-facts-label.

[48] FDA: Interactive Food Label Saturated Fat. https://www.accessdata.fda.gov/scripts/interactivenutritionfactslabel/saturated-fat.cfm.

[49] FDA: Interactive Food Label D Fiber. https://www.accessdata.fda.gov/scripts/interactivenutritionfactslabel/dietary-fiber.cfm.

[50] Lombard M.J., Steyn N.P., Charlton K.E., Senekal M. (2015). Application and interpretation of multiple statistical tests to evaluate validity of dietary intake assessment methods. Nutr. J..

[51] Masson L., Mcneill G., Tomany J., Simpson J., Peace H., Wei L., Grubb D., Bolton-Smith C. (2003). Statistical approaches for assessing the relative validity of a food-frequency questionnaire: Use of correlation coefficients and the kappa statistic. Public Health Nutr..

[52] Watson J.F., Collins C.E., Sibbritt D.W., Dibley M.J., Garg M.L. (2009). Reproducibility and comparative validity of a food frequency questionnaire for Australian children and adolescents. Int. J. Behav. Nutr. Phys. Act..

[53] Ma Y., Olendzki B., Pagoto S., Hurley T.G., Magner R.P., Ockene I.S., Schneider K.L., Merriam P.A., Hébert J.R. (2009). Number of 24-Hour Diet Recalls Needed to Estimate Energy Intake. Ann. Epidemiol..

[54] Shamah-Levy T., Rodríguez-Ramírez S., Gaona-Pineda E.B., Cuevas-Nasu L., Carriquiry A.L., A Rivera J. (2016). Three 24-Hour Recalls in Comparison with One Improve the Estimates of Energy and Nutrient Intakes in an Urban Mexican Population. J. Nutr..

[55] Carroll R.J., Midthune D., Subar A.F., Shumakovich M., Freedman L.S., Thompson F.E., Kipnis V. (2012). Taking Advantage of the Strengths of 2 Different Dietary Assessment Instruments to Improve Intake Estimates for Nutritional Epidemiology. Am. J. Eidemiol..

[56] DeBiasse M.A., Bowen D.J., Quatromoni P.A., Quinn E., Quintiliani L.M. (2018). Feasibility and Acceptability of Dietary Intake Assessment Via 24-Hour Recall and Food Frequency Questionnaire among Women with Low Socioeconomic Status. J. Acad. Nutr. Diet..

[57] NIDDK. https://www.niddk.nih.gov/health-information/kidney-disease/chronic-kidney-disease-ckd/eating-nutrition/nutrition-advanced-chronic-kidney-disease-adults.

[58] Aycart D., Acevedo S., Eguiguren-Jimenez L., Andrade J. (2021). Influence of Plant and Animal Proteins on Inflammation Markers among Adults with Chronic Kidney Disease: A Systematic Review and Meta-Analysis. Nutrients.

[59] Mirmiran P., Yuzbashian E., Aghayan M., Mahdavi M., Asghari G., Azizi F. (2020). A Prospective Study of Dietary Meat Intake and Risk of Incident Chronic Kidney Disease. J. Ren. Nutr..

[60] Haring B., Selvin E., Liang M., Coresh J., Grams M.E., Petruski-Ivleva N., Steffen L.M., Rebholz C.M. (2017). Dietary Protein Sources and Risk for Incident Chronic Kidney Disease: Results from the Atherosclerosis Risk in Communities (ARIC) Study. J. Ren. Nutr..

[61] Kim H., Caulfield L.E., Garcia-Larsen V., Steffen L.M., Grams M.E., Coresh J., Rebholz C.M. (2019). Plant-Based Diets and Incident CKD and Kidney Function. Clin. J. Am. Soc. Nephrol..

[62] Carrero J.J., González-Ortiz A., Avesani C.M., Bakker S.J.L., Bellizzi V., Chauveau P., Clase C.M., Cupisti A., Espinosa-Cuevas A., Molina P. (2020). Plant-based diets to manage the risks and complications of chronic kidney disease. Nat. Rev. Nephrol..

[63] Snelson M., Clarke R.E., Coughlan M.T. (2017). Stirring the Pot: Can Dietary Modification Alleviate the Burden of CKD?. Nutrients.

[64] Ball H.L. (2018). Conducting Online Surveys. J. Hum. Lact..

[65] Regmi P.R., Waithaka E., Paudyal A., Simkhada P., van Teijlingen E. (2016). Nepal Journal of Epidemiology Guide to the Design and Application of Online Questionnaire Surveys. www.nepjol.info/index.php/NJE.

