# Supplementary material for: Development and Relative Validity of the Chronic Kidney Disease Short Food Frequency Questionnaire (CKD SFFQ) to Determine Diet Quality and Dietary Habits among Adults with Chronic Kidney Disease
Source: Nutrients. 2021 Oct 15;13(10):3610. doi: 10.3390/nu13103610 (PMC8538370; doi:10.3390/nu13103610)
Supplement: Supplementary file 1 [file nutrients-13-03610-s001.zip › Supplemental table and figure.pdf]

**Supplemental Table S1. CKD SFFQ**

For the following food items listed, please use the following portion size guide based on measuring cups/spoons.

Grains and Starches (rice, pasta, bread): 1 cup, ½ cup cooked, 1 slice of bread
Fruit: Raw = 1 cup, Cooked/Canned = ½ cup
Vegetables: Cooked = 1 cup, Raw = 1 cup
Meat and alternatives (beans, tofu): 3 ounces cooked
Fats: 1 Tablespoon oil, 1/4 cup nuts
Beverages: Juices = ½ cup (4 ounces), Water/Milk = 1 cup (8 ounces)

Total Diet Score = 0 – 60 (0-30: low diet quality) (31-60: high diet quality)

**For the past 30 days, indicate how often you consumed the following portions of foods**

| **Foods/beverages** | **Daily** | **3-5 portions/week** | **1-2 portions/week** | **Rarely** | **Never** |
|---|---|---|---|---|---|
| White bread | | | | | |
| White pasta and rice | | | | | |
| Whole grain/ Wheat/ Brown bread | | | | | |
| Whole grain/ Brown: pasta and rice | | | | | |
| Whole grain breakfast cereals | | | | | |
| Non-whole grain breakfast cereals | | | | | |
| Legumes, beans | | | | | |
| Potatoes baked | | | | | |
| Potatoes roasted | | | | | |
| Potatoes mashed prepared with butter and milk | | | | | |
| Potatoes mashed prepared with water | | | | | |
| French fries | | | | | |
| Tomatoes: raw, cooked | | | | | |
| Vegetables (not counting potatoes): raw, cooked with no oil or salt | | | | | |
| Fruit raw: specify | | | | | |

| | | | | | |
|---|---|---|---|---|---|
| Canned fruit | | | | | |
| Dried fruit | | | | | |
| Soup – broth based | | | | | |
| Soup – cream based | | | | | |
| Pizza, lasagna, quiche | | | | | |
| Homemade; Sandwiches, burgers, burritos, tamales, enchiladas, tacos | | | | | |
| Processed non homemade; Sandwiches, burgers, burritos, tamales, enchiladas, tacos | | | | | |
| Fish: fried, breaded | | | | | |
| Fish: baked or grilled | | | | | |
| Seafood (other than fish; tuna, sardines) | | | | | |
| Chicken: fried, breaded | | | | | |
| Chicken: baked or grilled | | | | | |
| Red meat (beef, lamb, deer, camel): fried, breaded | | | | | |
| Red meat (beef, lamb, deer, camel): baked or grilled | | | | | |
| Pork: fried, breaded | | | | | |
| Pork: baked or grilled | | | | | |
| Sausages or other processed meats | | | | | |
| Eggs: boiled, scrambled, omelet prepared with fat | | | | | |
| Eggs: boiled, scrambled, omelet prepared without fat | | | | | |
| Milk, Yogurt: 2% or skim | | | | | |
| Milk, Yogurt: Whole or full fat | | | | | |

| | | | | | |
|---|---|---|---|---|---|
| Cheese soft (Mozzarella, Blue cheese, Feta) | | | | | |
| Cheese hard (Cheddar, Swiss) | | | | | |
| Cheese spread: cream | | | | | |
| Butter, sour cream | | | | | |
| Margarine, mayonnaise | | | | | |
| Mustard, salty condiments | | | | | |
| Ketchup, sweetened sauces (duck sauce) | | | | | |
| Olive oil | | | | | |
| Rapeseed oil, walnut oil, mixed oil | | | | | |
| Canola oil, sunflower oil | | | | | |
| Coconut oil | | | | | |
| Salty snacks | | | | | |
| Sweet cakes and snacks, chocolate and pastries | | | | | |
| Coffee | | | | | |
| Freshly brewed tea or herb teas | | | | | |
| Canned/ bottled tea or herb teas | | | | | |
| Sugar white, brown, cane | | | | | |
| Artificial sweeteners: Sweet'n low, Splenda, Equal, Stevia, etc | | | | | |
| Sweetened beverages (soda) | | | | | |
| Artificially sweetened beverages (diet soda) | | | | | |
| Water | | | | | |
| Wine | | | | | |
| Beer | | | | | |
| Other alcoholic beverages | | | | | |

**Supplemental Figure S1. Bland-Altman Plots to assess relative validity between CKD SFFQ and the 2-24-hour recalls**

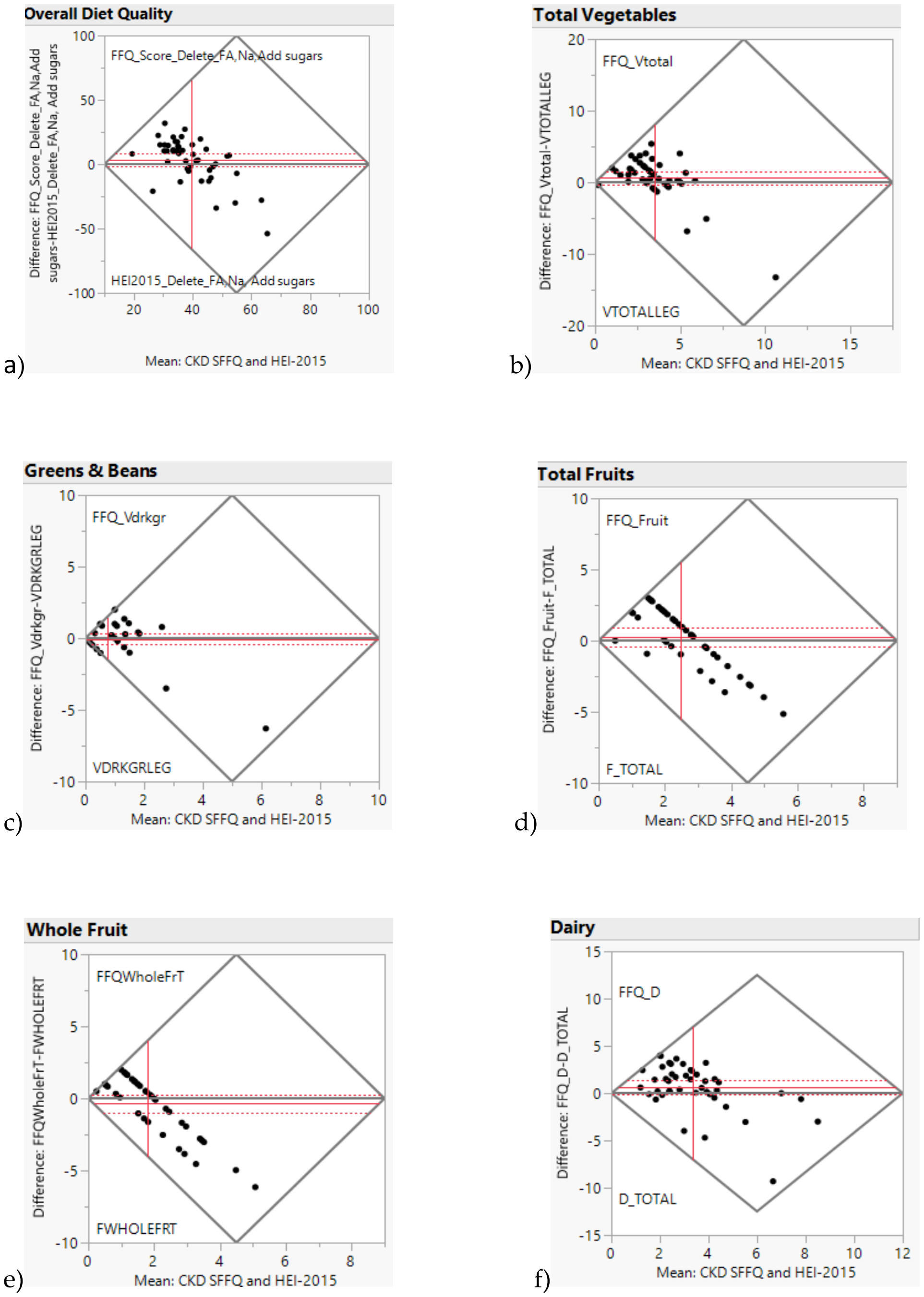

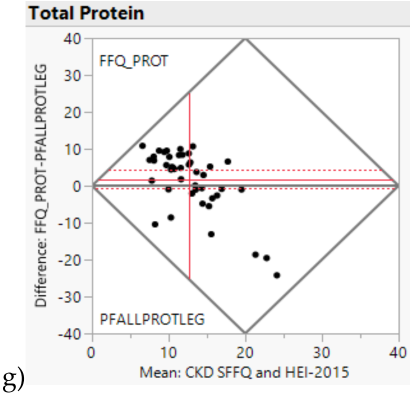


g)

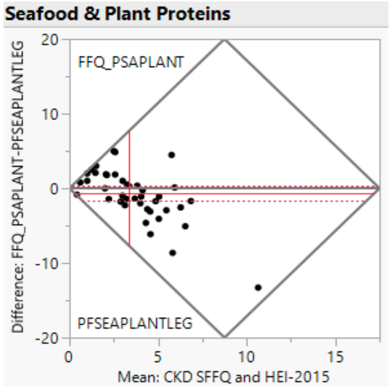


h)

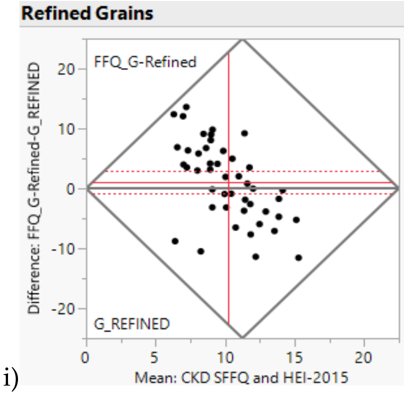


i)

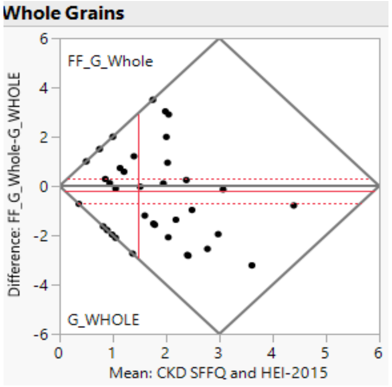


j)

*Figure S1.* Bland–Altman plots assessing the relative validity of the newly developed CKD SFFQ for estimating the diet quality of Adults, ≥18 years of age, who had CKD. For each participant, the difference in intake between the CKD SFFQ and the mean of 2-24-hour recalls (HEI-2015) is plotted against the mean intake from the two methods for: a) overall diet quality; b) total vegetables; c) green & beans; d) total fruit; e) whole fruit; f) dairy; g) total proteins; h) seafood & plant proteins; i) refined grains; and j) whole grains. Lines ——— represent the mean difference and lines - - - - - represent the 95 % limits of agreement. For overall diet quality scores, mean difference between the two methods was 3.19; for total vegetables, mean difference = -0.04; for greens & beans, mean difference = 0.63; for total fruits, mean difference = 0.25; for whole fruits, mean difference = -0.37; for dairy, mean difference = 0.64; for total proteins, mean difference = 1.71; for seafood & plant proteins, mean difference = -0.64; for refined grains, mean difference = 1.05; and for whole grains, mean difference = -0.20.
